# Predictors of Time to Effective and Optimal Antimicrobial Therapy in Patients With Positive Blood Cultures Identified via Molecular Rapid Diagnostic Testing

**DOI:** 10.1093/ofid/ofy350

**Published:** 2018-12-22

**Authors:** Maya Beganovic, Tristan T Timbrook, Sarah M Wieczorkiewicz

**Affiliations:** 1 Department of Pharmacy, Advocate Lutheran General Hospital, Park Ridge, Illinois; 2 Department of Pharmacy, University of Utah Health, Salt Lake City, Utah; 3 Salt Lake City Veteran’s Affairs Medical Center, Salt Lake City, Utah

**Keywords:** antimicrobial stewardship, bloodstream infections, metrics, predictors, rapid diagnostic testing

## Abstract

Antimicrobial stewardship (AMS) programs integrated with rapid diagnostic tests optimize patient outcomes and reduce time to effective therapy (TTET) and time to optimal therapy (TTOT). This study identifies predictors of TTET and TTOT among patients with positive blood cultures and identifies limitations to current TTOT definitions and outcomes.

Antimicrobial stewardship programs (ASPs) have emerged to combat the rapid rise of antimicrobial resistance, *Clostridioides difficile* infections, and other antimicrobial-related adverse events. These programs employ Infectious Diseases Society of America (IDSA) guideline-supported tools including rapid diagnostic tests (RDTs) to improve patient outcomes through early organism identification and prompt antimicrobial optimization [1]. Although RDTs provide essential information, a meta-analysis indicates that this technology may not be as impactful without ASPs [2]. Furthermore, antimicrobial stewardship (AMS) team response to RDT results can lead to faster time to effective therapy (TTET) and time to optimal therapy (TTOT) and is associated with reduced mortality, length of stay, and bacteremia recurrence [3]. However, studies delineating predictors of TTET and TTOT are limited; therefore, this study aims to identify such predictors.

## METHODS

### Study Setting and Design

This cohort study was conducted at a 645-bed tertiary care teaching hospital between November and February of 2014–2015 and 2015–2016. These time periods were chosen to reflect standard AMS practices provided in a comparative time frame to implementation of real-time AMS in November 2015. In 2014 and early 2015, the AMS team conducted daily, non-real-time prospective audit and feedback on all patients with positive blood cultures (BCs). Although our AMS team was multidisciplinary and approved the protocols utilized in this study, the pages were largely received by a pharmacy resident under the direct supervision of an infectious diseases pharmacy specialist. Adult and pediatric patients with at least 1 positive BC were evaluated. Only the first positive BC per patient was included. Patients were assessed via retrospective chart review and excluded if they expired before BC result or if transferred from another hospital with active bacteremia.

### Microbiology Laboratory Procedures

Blood cultures were analyzed for the presence of microorganisms every 2 hours using the BacT/ALERT Microbial Detection System (bioMérieux), as previously described [4]. Once a BC is positive, it is Gram-stained and rapidly identified by matrix-assisted laser desorption ionization–time of flight (MALDI-TOF). Gram stain results were called to the nurse; however, MALDI-TOF results were posted in patients’ medical charts by the microbiology laboratory without notification until the implementation of the real-time AMS team, which responded to all positive BCs. After this implementation, positive BCs identified by MALDI-TOF were communicated via AMS pager 24 hours per day, 7 days per week [4]. With the exception of patients requiring immediate attention (eg, bug-drug mismatches), pages received between 22:00 and 06:00 were addressed the following morning. For patients with an infectious diseases consult (IDC), AMS recommendations were made to the IDC team.

### Outcome Definitions

Predictors of TTOT and TTET were analyzed and measured from BC collection. Optimal therapy was defined as the most appropriate treatment option for a given organism and source of bacteremia utilizing an ASP-approved bacteremia guideline [4]. This may include escalation, de-escalation, or discontinuation of duplicative or unnecessary (eg, noninfectious etiology or contaminated culture) therapy. Cultures growing Gram-positive skin commensals suspected of contamination were assessed for several factors, including number of positive BCs, presence of a central venous catheter, prosthetic heart valve or other clinical risk, and patient clinical stability. For instance, clinically stable patients with 1 BC bottle growing coagulase-negative staphylococci in the absence of indwelling catheters or devices were deemed contaminants. Effective therapy was defined as any regimen active against the causative pathogen, as defined by culture susceptibility. Contaminated cultures were excluded from analysis in TTET. Additional descriptive characteristics of the overall cohort were collected, including in-hospital all-cause mortality, hospital and intensive care unit length of stay, time to microbiologic clearance from initial blood culture draw, antimicrobial length of therapy (including inpatient and planned outpatient treatment duration), 30-day bacteremia recurrence, infectious diseases (ID) consult, and time to ID consult. This study was considered a quality improvement project and was exempt from institutional review board review.

### Statistical Analysis

Manual stepwise multiple linear regression models were utilized with TTOT and TTET as the dependent variables, and the following explanatory variables: presence of real-time AMS team response, age, sex, clinical status (ie, general floor vs intensive care unit admission), acquisition (ie, hospital- vs community-acquired infection), severity of illness, comorbidities, source of bacteremia, organism type (eg, Gram-positive, Gram- negative, yeast), and presence of multidrug-resistant organisms (MDROs). Variables significant at an alpha of .05 were retained in the final models. The analyses were performed using R studio software (R Foundation for Statistical Computing, Vienna, Austria).

## RESULTS

A total of 239 patients were included in the final analysis: 183 with bacteremia or candidemia and 56 contaminants. Most BC-positive organisms were Gram-positive (54.4%), with coagulase-negative *Staphylococcus* (CoNS; 46.1%), *Streptococcus* spp. (19.2%), and methicillin-sensitive *Staphylococcus aureus* (16.1%) representing the most commonly observed isolates. This was followed by Gram-negative organisms (40.6%), with *E. coli* (49.5%) and *Klebsiella* spp. (10.3%) representing the majority. The most common sources of bacteremia included urinary tract (27%), intra-abdominal (19%), respiratory (13%), and unknown (13%). Cohort characteristics are detailed in Table 1.

**Table 1.** Characteristics of Cohort With Positive Blood Culture Results

Characteristic	No. (%)^a^(n = 239)
Age, median (IQR), y	67 (52–80)
Male sex	110 (46)
Pitt Bacteremia Score, median (IQR)	2 (0–4)
Charlson Comorbidity Index, median (IQR)	5 (3–7.5)
Acquisition type	
Community	112 (46.8)
Hospital	73 (30.5)
Unknown	54 (22.6)
Clinical status	
General medicine	159 (66.5)
Intensive care unit	80 (33.5)
Real-time AMS team response	123 (51.4)
MDRO presence	48/183 (26.2)
Contaminants	56 (23.4)
TTET, median (IQR), h^b^	6.75 (1.95–23.07)
TTOT, median (IQR), h	41.89 (8.595–73.18)
Time to first negative culture, median (IQR), h^b^	42.75 (27.8–57.32)
ID consult	175 (73.2)
TTIDC, median (IQR), h	18.02 (4.59–25.25)
HLOS, median (IQR), d	7 (4–14)
ICU LOS, d	1.95 (0.2–6.7)
Antimicrobial length of therapy, median (IQR), d	14 (12–18)
In-hospital mortality	27 (11.3)
30-d recurrent bacteremia^d^	6 (3.4)
Source of infection^b^	(n = 183)
Urinary tract infection/pyelonephritis	49 (27)
Intra-abdominal infection	34 (18.6)
Respiratory tract infection	24 (13)
Line/port infection	17 (9.3)
Febrile neutropenia	3 (1.6)
Endocarditis	3 (1.6)
Other	53 (28.9)

Abbreviations: AMS, antimicrobial stewardship; HLOS, hospital length of stay; ID, infectious diseases; ICU LOS, intensive care unit length of stay; IQR, interquartile range; MDRO, multidrug-resistant organism; TTET, time to effective therapy; TTIDC, time to infectious diseases consult; TTOT, time to optimal therapy.

^a^Unless otherwise specified.

^b^Total n = 183 (contaminants excluded from analysis).

^c^In-hospital mortality was 10.4% when excluding contaminated cultures (n = 183).

^d^Total n = 116 (contaminants excluded from analysis; 67 patients lost to follow-up).

**Figure 1.**
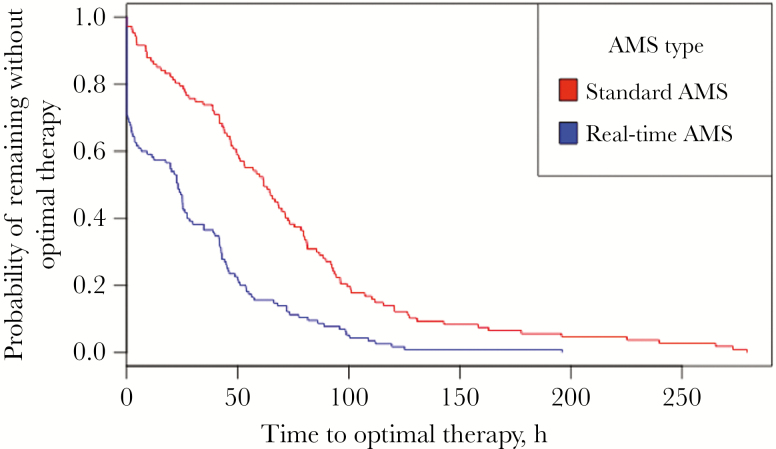
Time to optimal therapy by type of antimicrobial stewardship (AMS) intervention.

Real-time AMS team response (beta coefficient, –34.18; 95% confidence interval [CI], –47.6 to –20.8) was identified as the only independent predictor of shorter TTOT and was associated with a significantly higher probability of receiving optimal therapy when compared with standard AMS intervention during the pre-implementation period (*P *< .05) (Figure 1). Having febrile neutropenia (beta coefficient, 93.39; 95% CI, 38.1 to 148.7) or endocarditis (beta coefficient, 60.06; 95% CI, 4.8 to 115.3) was associated with a longer TTOT.

Independent predictors of faster TTET included having urinary tract infections or pyelonephritis as the source of bacteremia (beta coefficient, –10.7; 95% CI, –19.1 to –2.3) and being infected with a Gram-positive organism (beta coefficient, –7.8; 95% CI, –13.6 to –0.98). On the contrary, longer TTET was predicted by presence of MDRO (beta coefficient, 7.12; 95% CI, 1.4 to 12.9) and bacteremia consequent to surgical site infections (beta coefficient, 17.21; 95% CI, 1.2 to 33.3).

## DISCUSSION

Our study identified real-time AMS team response as an independent predictor of faster TTOT when compared with standard AMS practices of daily blood culture review (Figure 1). Antimicrobial practices (eg, selection of empiric antimicrobials) did not change between the 2 periods; however, receiving real-time pages and promptly responding resulted in a significant difference in time to optimal therapy early in the patient’s treatment course, whereas before AMS team response, adjustments were made after 48–72 hours as providers were unaware of the rapid organism identification. However, real-time AMS team response did not impact TTET, likely because TTET is related to empiric treatment selection, and most patients are initiated on broad-spectrum antimicrobials. This also explains why urinary tract infections and Gram-positive organisms are predictors of faster TTET, as most patients are started on agents that cover typical urinary isolates (eg, empiric cefepime, piperacillin/tazobactam) and Gram-positive organisms (eg, empiric vancomycin).

Real-time AMS team response expediting TTOT is supported by randomized controlled trials, which show that the addition of real-time AMS intervention resulted in earlier active and appropriate therapy [5], resulted in faster de-escalation, and eliminated or reduced the treatment of contaminants [6]. Integration of AMS intervention and RDT was also associated with reduction in TTOT and mortality in patients with MDROs, with AMS intervention being an independent predictor of survival [7]. Although delayed TTET was predicted by the presence of MDROs, TTOT was not, potentially due to rapid streamlining by our AMS team. Nonetheless, therapy adjustments for MDROs without antimicrobial susceptibility results may be challenging; therefore, RDTs with genotypic resistance testing can be useful and previously demonstrated a reduction in TTET for extended-spectrum beta-lactamase–producing organisms [8].

We identified endocarditis and febrile neutropenia as predictors of prolonged TTOT, likely due to provider reluctance in de-escalating therapy for infections associated with high mortality risk. These findings are corroborated by a recent study evaluating RDT in addition to AMS in cancer patients with CoNS-positive BCs, where neutropenia was independently associated with prescription of antimicrobials (odds ratio, 2.76; 95% CI, 1.06 to 7.18) [9]. These findings indicate that more data are required to support de-escalation or discontinuation among cancer patients. Furthermore, this suggests that including these patients in the calculation of TTOT, and potentially other metrics evaluating antimicrobial utilization, may skew overall results and requires consideration when analyzing AMS data.

In addition to reluctance to de-escalate in high-mortality infections, a recent survey evaluating physician interpretation of BCs revealed that overall misinterpretation can be as high as 50% [10], an unfortunate finding given that de-escalation to optimal therapy is associated with improved patient outcomes [3] and cost savings [11]. However, provider satisfaction with real-time AMS clinical decision support has been reported to be high (4.8/5-point Likert scale) with respect to provider autonomy and appropriateness of recommendation [12], demonstrating and supporting well-received value in real-time AMS team response. Our study is limited by a small sample size and observational design lacking randomization. Although our data support a clear incremental benefit of integrating real-time AMS response with RDT, they also suggest that patients with severe disease and infections associated with immunosuppression, specifically febrile neutropenia, may prolong TTOT and require further attention as the safety of rapid streamlining in this population has yet to be determined and AMS metrics remain to be standardized.

## Notes

Prior presentation. Preliminary results of this work were presented at the 28th ECCMID on April 22, 2018, in Madrid, Spain, poster # P0908.
